# A Safety and Efficacy Study of a Synthetic Biolubricant in an Equine Model of Post-Traumatic Osteoarthritis

**DOI:** 10.3390/ani15030404

**Published:** 2025-02-01

**Authors:** Lauren K. Luedke, Kathryn A. Seabaugh, Benjamin G. Cooper, Brian D. Snyder, Markus A. Wimmer, C. Wayne McIlwraith, Myra F. Barrett, Christopher E. Kawcak, Mark W. Grinstaff, Laurie R. Goodrich

**Affiliations:** 1 Gail Holmes Equine Orthopaedic Research Center, Translational Medicine Institute, Department of Clinical Sciences, College of Veterinary Medicine and Biomedical Sciences, Colorado State University, Fort Collins, CO 80523, USA; lauren.luedke@colostate.edu (L.K.L.); katie.seabaugh@colostate.edu (K.A.S.); wayne.mcilwraith@colostate.edu (C.W.M.); myra.barrett@colostate.edu (M.F.B.); christopher.kawcak@colostate.edu (C.E.K.); 2 Departments of Chemistry and Biomedical Engineering, Boston University, Boston, MA 02215, USA; bgcooper@bu.edu (B.G.C.); mgrin@bu.edu (M.W.G.); 3 Department of Orthopedic Surgery, Boston Children’s Hospital, Boston, MA 02115, USA; brian.snyder@childrens.harvard.edu; 4 Department of Orthopedic Surgery, Rush University, Chicago, IL 60612, USA; markus_a_wimmer@rush.edu

**Keywords:** osteoarthritis, viscosupplementation, biolubricant, horses

## Abstract

Post-traumatic osteoarthritis (PTOA) is a common cause of lameness in the horse. There is no cure, therefore treatments are aimed at reducing pain and improving the joint environment by modifying inflammatory pathways or by viscosupplementation. Here, we report the safety and efficacy of the biolubricant (poly(2-methacryloyloxyethyl phosphorylcholine; pMPC) to mitigate the physical, gross, histological, and biochemical effects of arthritis. We created an osteochondral fragment in the middle carpal joint of one limb in 16 horses to induce PTOA; the contralateral limb served as a sham-operated joint. Two weeks postoperative, half (n = 8) of the horses received a single injection of pMPC in the PTOA joint, while the other half received saline. All sham-operated joints (n = 16) received saline. We conducted clinical evaluations weekly while synovial fluid biomarkers were measured biweekly during the 70-day study period. Subsequently, we performed postmortem gross and histologic analyses. Horses in which PTOA joints were treated with pMPC exhibited mild increases in clinical data, including lameness, effusion, and flexion scores. Similarly, synovial cell count, total protein, and prostaglandin E_2_ values were higher for pMPC-treated joints. Radiographic changes included significantly higher osteophyte scores in pMPC-treated joints at the terminal timepoint. The biolubricant may demonstrate some chondroprotective effects with lower total erosion scores and higher cartilage glycosaminoglycan content. In summary, when pMPC is administered to PTOA joints, the biolubricant induces a mild inflammatory response but may offer some chondroprotective effects in horses.

## Simple Summary

The biolubricant polymer, poly(2-methacryloyloxyethyl phosphorylcholine) (pMPC), enhances articular lubrication in ex vivo cartilage explants and protects articular cartilage in an in vivo small animal model. Herein, we assessed the efficacy of the biolubricant pMPC for the treatment of a highly translational model of post-traumatic osteoarthritis (PTOA) model in horses. The pMPC may demonstrate cartilage protective effects with lower total cartilage erosion scores and increased levels of glycosaminoglycan retained in the cartilage compared to saline-treated joints. However, a mild inflammatory state is present on a cellular level, resulting in clinical evidence of mild pain scores and increased osteophyte formation. Further research is warranted to elucidate a chemical structure, concentration, and dose that maximizes the cartilage protective effects and minimizes the synovial inflammatory effects.

## 1. Introduction

Post-traumatic osteoarthritis (PTOA) is a painful, protracted, degenerative disease of joints that affects an estimated 3 million horses and 250 million people worldwide [1,2]. It is reportedly the most common cause of lameness in horses, with significant economic impact due to costs associated with detection and treatment of PTOA [3]. The disease process has not been completely elucidated; however, it is evident that the upregulation of catabolic pathways results in prolonged inflammation and, ultimately, degradation of the articular cartilage [4]. There is no cure for PTOA, and current treatments are focused on relieving the pain via inhibiting inflammatory pathways (e.g., nonsteroidal anti-inflammatory drugs) or via viscosupplementation (e.g., synthetic hyaluronate). Treatment also focuses on modifying the disease state by reducing gross articular cartilage degeneration.

Pharmacological interventions that treat the underlying disease have been widely investigated and include small molecule, protein, and gene therapies, with many in pre-clinical large animal studies [5,6,7,8,9]. An alternative strategy is one that uses a material to improve the joint environment. One such example is the use of an aqueous polymer solution injected intra-articularly to re-establish synovial fluid viscosity and lubricity, thereby providing chondroprotection [10,11,12,13,14]. Inspiration for these biolubricants comes from mimicking the lubricating effects of endogenous bioproteins such as hyaluronic acid, mucin, and lubricin [9,15,16,17]. Examples include polyacrylates [polyacrylic acid, ref poly(acryloylamino-2-methyl-1-propanesulfone), poly(2-methacryloyloxyethyl phosphorylcholine and poly(2-methacryloyloxyethyl phosphorylcholine)]; polyolefins [poly(7-oxanorbornene-2-carboxylate)], polyamides (polylysine grafted HA), and polyacrylamide hydrogels (PAAG) [9,18,19,20,21,22,23,24,25]. Enhanced lubrication in the joint is advantageous as it dissipates shear forces on articular cartilage, thereby reducing wear from repetitive joint loading [9]. While the end goal of many synthetic biolubricants is the same, the chemical formulation, physical properties, manufacturing, and tissue interaction can be highly variable and therefore are not always directly comparable [26].

One of these biolubricants, poly(2-methacryloyloxyethyl phosphorylcholine) (pMPC), is zwitterionic in nature-equal positive and negative electrical charges and when coated on a surface results in a hydrophilic, low-friction material [9]. The pMPC functions to augment the extracellular matrix of cartilage. Application of pMPC, either in a linear or crosslinked format, to ex vivo bovine cartilage explants reduces the coefficient of friction and decreases tissue strain compared to saline and is superior to hyaluronic acid [9]. In a model of repeated administration, the crosslinked pMPC outperforms the linear pMPC in its ability to reduce the coefficient of friction and cushion ex vivo cartilage surfaces. Furthermore, a sustained duration of effect was demonstrated, with a prolonged intra-articular residence time of over 30 days in a rat model for PTOA [22]. Herein, we report the safety and efficacy of an intra-articular pMPC in an established model of PTOA in the horse. We hypothesized the pMPC would decrease the physical, gross, radiographic, histological and biochemical effects of PTOA in this highly translational model of PTOA.

## 2. Materials and Methods

### 2.1. Horses

Sixteen horses were included in the study. The horses consisted of Quarter Horses or mixed-breed Quarter Horses; were of a mixed population of mares and geldings; and ranged in age from 2 to 5 years old. Pre-study evaluations included general health, subjective lameness examination, evaluation of carpal effusion, and carpal radiographs. To be admitted to the study, horses required a lameness score of less than or equal to 1 out of 5 on a straight line [American Association of Equine Practitioners’ (AAEP) lameness scale [27], 0 (normal gait) to 5 (non-weight bearing lameness)] and radiographically normal carpi. Horses were acclimatized to exercise on a high-speed treadmill over the course of 14 days prior to surgery. The study was approved by the institution’s animal care and use committee (Protocol 15-6239A).

### 2.2. Synthetic Biolubricant

The polymeric lubricant used was 5 w/v% poly(2-methacryloyloxyethyl phosphorylcholine) (pMPC) [9]. The network polymer was synthesized via copolymerization with the crosslinker ethylene glycol dimethacrylate maintained at 1 mol% (mol/mol MPC). The polymers were purified via dialysis, lyophilized, and resuspended in deionized water at 5 w/v%.

### 2.3. Experimental Induction of Osteoarthritis

Following induction of anesthesia and routine preparation for bilateral carpal arthroscopy, one middle carpal joint was randomly selected for the surgical induction of OA while the other served as a sham-operated control joint (day 0). Surgeries were performed using a standard approach and a previously described model [5]. Briefly, an 8 mm osteochondral fragment was created on the distal aspect of the radial carpal bone at the level of the synovial plica and left in situ attached to the joint capsule. The fragment gap was widened to 15 mm using an arthroscopic burr (Arthrex, Munich, Germany). The fragment and all debris from the burring were not removed from the joint [5,6]. In the sham-operated joint, the absence of significant lesions was confirmed.

All horses were preoperatively treated with Cefazolin (11 mg/kg IV) and Gentamicin (6.6 mg/kg IV). Phenylbutazone was administered at 4.4 mg/kg per os (PO) preoperatively and continued once daily for an additional 2 days.

### 2.4. Treatments

**Figure 1:**
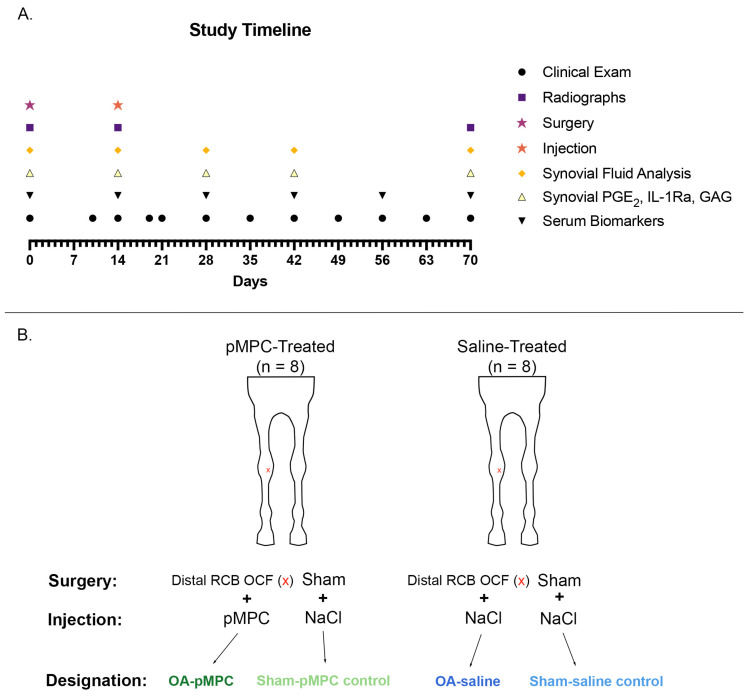
(**A**) Study timeline in days. (**B**) The study design consisted of 16 horses in total, including 8 horses in the polyacrylate (pMPC)-treated group, and 8 horses in the saline-treated group. For each horse, one randomly selected limb (n = 8 left, n = 8 right) was designated for the induction of OA by creating an osteochondral fragment (OCF) in the distal aspect of the radial carpal bone (RCB) as indicated by the red X; the other limb was sham-operated. On day 14 postoperatively, each OA limb was injected with either 6 mL pMPC (n = 8) or 6 mL saline (n = 8); all sham-operated limbs were injected with 6 mL saline (n = 16).

Twelve days after surgery, the horses were evaluated for lameness and designated to one of two groups (OA–treatment vs. OA–control). To equalize the lameness grades per group, they were ranked based on lameness scores and randomly designated to each group by alternating the designation. Eight horses were assigned to the biolubricant (pMPC) treatment group and eight to the untreated group (Figure 1). On day 14, 1.25 mL of synovial fluid was collected from each horse in the treatment group via arthrocentesis from each middle carpal joint. Subsequently, the OA joint received an intra-articular injection of 6ml pMPC, while the contralateral sham-operated joint received 6 mL 0.9% saline intra-articularly. In the untreated group, similarly, each middle carpal joint had 1.25 mL of synovial fluid aspirated via arthrocentesis, followed by an intra-articular injection of 6 mL saline. The injector (LRG) was not blinded to treatment nor OA-induced joints. Horses were administered a 1.1 mg/kg dose of flunixin meglumine intravenously once daily for 3 days.

### 2.5. Exercise Protocol

Horses were housed individually in 3.65 × 3.65 m stalls. Days 1–12, horses were maintained on stall rest. Day 13 and again on Day 14 prior to treatment, the horses were exercised lightly at a trot (4.0–5.0 m/s) for 6 min on a high-speed treadmill (EquiGym, Lexington, KY, USA). The horses were allowed 4 days of rest following treatment (days 15–18).

### 2.6. Lameness Scores

Clinical examination and lameness evaluations were performed by a board-certified equine sports medicine specialist (KAS) unaware of the treatment groups. Baseline lameness was assessed following the initial treadmill acclimatization period and reported prior to surgery on day 0. Postoperative lameness exams were performed on days 10, 14, and 19 (prior to, day of, and following treatment) and then once weekly starting on day 21 until day 70. Subjective and objective lameness data were reported for each lameness evaluation. The subjective evaluation used the AAEP 1–5 graded lameness scale [27]. Objective lameness data were collected using an inertial sensor system (Equinosis^®^ Lameness Locator, Columbia, MO, USA) [28]. Specifically, the foresigned vector sum was documented, which indicates the direction (positive values for right forelimb lameness and negative values for left forelimb lameness) and magnitude of lameness calculated from millimeters of displacement. Clinical lameness is associated with a vector sum greater than 8.5 mm. At each lameness exam, horses were also evaluated for carpal effusion and response to flexion using a subjective ordinal grading scale of 0 to 4 (0 = normal, 1 = slight, 2 = mild, 3 = moderate, and 4 = marked/severe).

### 2.7. Diagnostic Imaging

Radiographic assessment of both carpi was performed prior to study inclusion (baseline; day 0), day 14, and day 70. Radiographic views included lateromedial, dorsopalmar, dorso 30° medial–palmarolateral oblique (DMPLO), dorso 45° lateral–palmaromedial oblique (DLPMO), and flexed lateromedial projections. A board-certified radiologist (MBF), blinded to treatment grouping, graded the radiographic examinations based on a previously established grading scale for five parameters: (1) osseous proliferation at the dorsal joint capsule (enthesopathy), (2) subchondral bone lysis of the radial carpal bone, (3) subchondral sclerosis of the radial carpal bone, (4) subchondral sclerosis of the third carpal bone, and (5) osteophyte formation. For each radiographic outcome parameter, a scale of 0 to 4 was used (0 = no detectable abnormality, 1 = slight change, 2 = mild change, 3 = moderate change, and 4 = severe change). A total radiographic score was also calculated for each limb based on the summation of scores from the 5 parameters.

### 2.8. Synovial Fluid Analysis

Synovial fluid was collected from each middle carpal joint on days 0, 14, 28, 42, and 70; approximately 1.25 mL was aspirated at each collection. Half of this volume was analyzed for total nucleated cell count and total protein concentrations (within 12 h of collection); the other half was centrifuged, and the supernatant was frozen at −80 °C in plastic microtubes until analysis for prostaglandin-E2 (PGE_2_); interleukin 1 receptor antagonist protein (IL-1Ra) and glycosaminoglycan (GAG) concentrations were performed as previously described [29,30].

### 2.9. Serum Biomarkers

Whole blood was harvested from the jugular vein and processed to harvest serum on days 0 and 14 and then once every other week from days 28–70. An aliquot of serum was used to measure liver and kidney function enzymes, including Aspartate Aminotransferase (AST), Gamma-glutamyl Transferase (GGT), and Creatinine (within 12 h of collection). The remaining serum was frozen at −80 °C in plastic microtubes until PGE_2_ and GAG concentrations were analyzed.

### 2.10. Gross Pathology and Histology

Horses were euthanized with an overdose of intravenous sodium pentobarbital (Euthanasia Solution, VetOne, Boise, ID, USA). Immediately following euthanasia, their middle carpal joints were disarticulated, photographed, and evaluated for the presence/absence of fragments, fragment size, total cartilage erosion, total joint hemorrhage, full thickness and partial thickness cartilage erosion, kissing lesions, and synovial adhesions [5]. All parameters were graded on a subjective ordinal scale (0 = normal to 4 = severe) apart from the kissing lesions and synovial adhesions, which were graded based on their presence (yes or no) [5]. The expert grading the joints (KAS) was not blinded to the presence of osteochondral fragments but was blinded to treatment group assignments.

Following gross macroscopic evaluation, synovium, cartilage, and subchondral bone specimens were collected. Synovium was harvested from a villous area. Cartilage was harvested from the radial facet of the third carpal bone (C3), the fourth carpal bone (C4), and the distal radial carpal bone (RCB); subchondral bone samples were harvested from C3 and RCB. All samples were placed in neutral-buffered 10% formalin and processed for histologic evaluation. Cellular changes were assessed in synovial, cartilage, and subchondral bone samples using Hematoxylin and Eosin (H and E) staining. Synovium was evaluated for intimal hyperplasia, subintimal edema, subintimal fibrosis, and vascularity. Cartilage was evaluated for fibrillation, chondrone formation, chondrocyte necrosis, and focal cell loss. Subchondral bone was evaluated for osteochondral lesions, subchondral bone remodeling, and osteochondral splitting. Changes in GAG content were assessed in cartilage by staining with Safranin O and Fast Green (SOFG). For each of the previously mentioned samples, GAG content was measured as the presence of stain uptake in tangential, intermediate, radiate territorial, and radiate interterritorial zones. For all synovium, cartilage, and subchondral bone samples, individual scores were assigned as well as a summation score for all parameters. Histology was graded by a single evaluator (LRG) blinded to the treatment assignments using a modified Mankin scoring system [31].

### 2.11. Surface Topography

Surface topography was performed as previously described [32]. In short, osteochondral plugs 4 mm × 4 mm × 8 mm were harvested from C3 and sent to the Department of Orthopedic Surgery, Rush University Medical Center for surface topography analysis. Using a scanning white light interferometry microscope, nine measurements were taken from the surface of each sample in a 3 × 3 square array. Mean parameters were computed for the following: maximum peak-to-valley depth (PV), root mean square roughness (Rq), arithmetic mean roughness (Ra), skewness (Rsk)—a measure of the symmetry of the deviations about the center plane—and the arithmetic average of the five highest peaks and five lowest valleys (SRz).

### 2.12. Statistical Analysis

Statistical analysis was performed using SAS 9.4. Residual diagnostic plots were used to evaluate assumptions of normality and equal variance. Some variables were transformed (using log or square root) in order to better satisfy model assumptions.

A mixed model was run separately for each response variable. All models included horse, within treatment, and phase as random effects. Seventeen variables were identified as primary responses, including lameness (subjective and objective), flexion, and effusion scores for clinical analysis; osteophytes and summation scores for radiographic analysis; IL-1Ra, PGE_2_, total protein, and total nucleated cell count for synovial fluid analysis; cartilage summation, fibrillation, full thickness, partial thickness, and total erosion scores; and synovial subintimal fibrosis and summation scores for gross and histologic analysis. For the additional response variables, a Bonferroni adjustment (corresponding to the other 66 responses) was used to control for multiple testing. If there was evidence of a treatment main effect or interaction based on F-tests, then pairwise comparisons were considered.

Clinical analysis, radiographic analysis, and synovial fluid analysis were measured for each limb at the aforementioned timepoints (Figure 1). Treatment, OA status, day, and all interactions were included as fixed effects. At each timepoint, the four combinations of treatment and OA were compared using Tukey’s method. For each treatment/OA combination, comparisons versus day 14 (postoperative, prior to injection) were made using Dunnett’s method.

Serum biomarkers were measured for each animal at the aforementioned timepoints (Figure 1). Treatment, day, and treatment*day interaction were included as fixed effects.

Gross pathology, histology, and surface topography were all measured on each limb at the final timepoint. Treatment, OA status, and treatment * OA interaction were included as fixed effects. The four combinations of treatment and OA were compared using Tukey’s method.

## 3. Results

### 3.1. Horses

A total of 16 mixed-breed horses were accepted into the study: eight mares and eight geldings, with a mean age of 2.5 years (range 2–4 years) and a mean weight of 379 kg (range 322–443 kg). The treatment group comprised five mares and three geldings, with a median age of 2.5 years and a median weight of 400 kg. In the control group, the eight horses consisted of three mares and five geldings, with a median age of 2.5 years and a median weight of 369 kg.

### 3.2. Clinical Examination

#### 3.2.1. Lameness Scores

**Figure 2:**
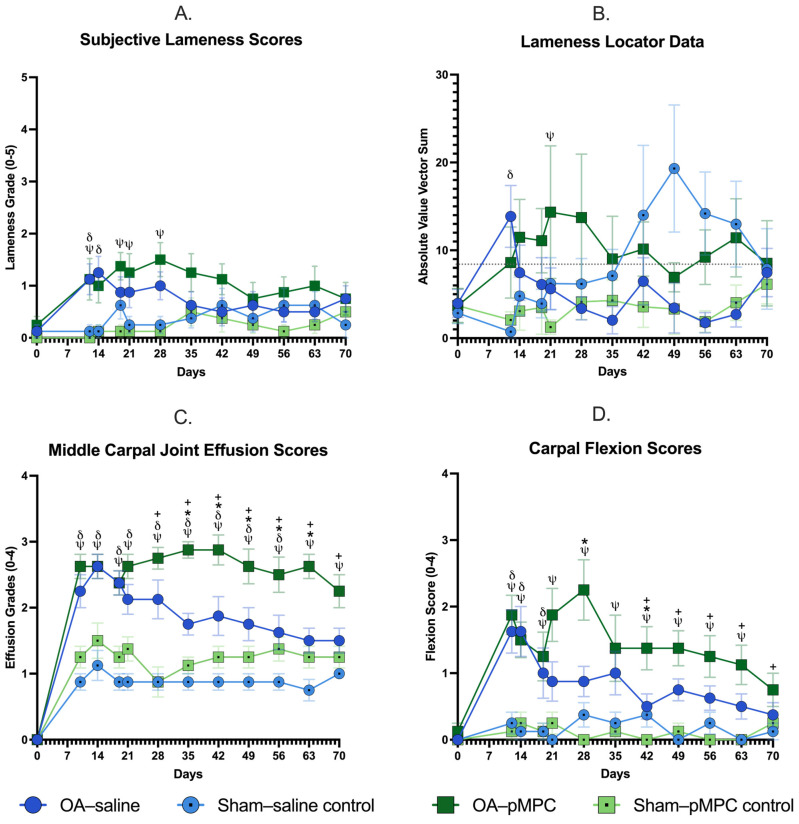
Clinical data with (**A**) subjective lameness scores, (**B**) lameness locator data, (**C**) middle carpal joint effusion scores, and (**D**) carpal flexion scores for each forelimb throughout the study period. The mean +/− SEM is represented at each evaluation timepoint. Surgery is represented by day 0, and injections were performed on day 14. For all graphs, *ψ* represents days for which OA–pMPC limbs had significantly greater lameness/effusion/flexion scores than their sham-operated counterpart (Sham–pMPC control); δ represents days for which OA–saline limbs had significantly greater lameness/effusion/flexion scores compared to their sham-operated counterpart (Sham–saline); * represents days OA–pMPC had significantly different effusion/flexion scores than OA–saline limbs; and + represents days effusion/flexion scores were significantly lower than baseline day 14 scores for OA–saline limbs. For lameness locator data, clinical lameness is considered for values greater than 8.5 mm, as indicated by the dotted line in (**B**).

Subjective lameness scores were mild overall, consistently graded as 1 ± 0.5 throughout the study following surgery (Figure 2). Limbs with induced OA were significantly more lame than their sham-operated counterpart postoperatively for both groups. OA–pMPC limbs had a more sustained level of lameness (*p* < 0.02 for all noted timepoints in Figure 2A,B). For all limbs, lameness scores were below 2 on the 4-point scale and not significantly different from pretreatment baseline (D14) at any timepoint. Objective lameness scores were not significantly different for OA–pMPC versus OA–saline limbs at any timepoint. There were no significant differences for any limbs using the lameness locator data at any timepoint (Figure 2B).

#### 3.2.2. Carpal Effusion

Effusion scores increased following surgery for all limbs (Figure 2C) and remained mild to moderately elevated. Effusion scores for OA–pMPC limbs were significantly higher than the sham-operated limb for the entire postoperative duration of the study, while OA–saline limbs had significantly higher effusion scores through day 56 (*p* < 0.005 for all timepoints indicated in Figure 2). Initially, there was no significant difference between effusion scores for OA–pMPC versus OA–saline; however, effusion scores for OA–saline limbs began to decrease while OA–pMPC limbs remained persistently elevated throughout the study. On days 28–70, effusion scores were significantly lower for OA–saline limbs from the pretreatment baseline (d14) (*p* < 0.001 for days 35–70), while OA–pMPC limbs did not change throughout the study. Effusion scores were significantly higher for OA–pMPC compared to OA–saline on days 35–63 (*p* < 0.02 for all timepoints indicated in Figure 2).

#### 3.2.3. Response to Flexion

Flexion scores were overall mild for both groups (Figure 2D). Postoperatively, OA–pMPC limbs had flexion scores that remained significantly higher than Sham–pMPC control limbs through day 63 (*p* < 0.003 for all timepoints, Figure 2D), while OA–saline limbs were significantly different from sham–saline control limbs through day 19 (*p* < 0.04 for all timepoints, Figure 2D). There was no significant difference between OA–pMPC- and OA–saline-treated limbs 7 days following treatment; however, flexion scores for OA–saline-treated limbs progressively decreased while OA–pMPC flexion scores remained elevated. For OA–pMPC limbs, the decrease was never significantly different from d14; however, flexion scores for OA–saline limbs were significantly lower on days 42–70 compared to d14 (*p* < 0.01 days 42–70). When comparing between treatments, flexion scores were significantly higher for OA–pMPC limbs on days 28 and 42 than for OA–saline limbs (*p* = 0.0002, *p* = 0.04, respectively).

### 3.3. Synovial Fluid Analysis

#### 3.3.1. Total Nucleated Cell Count

**Figure 3:**
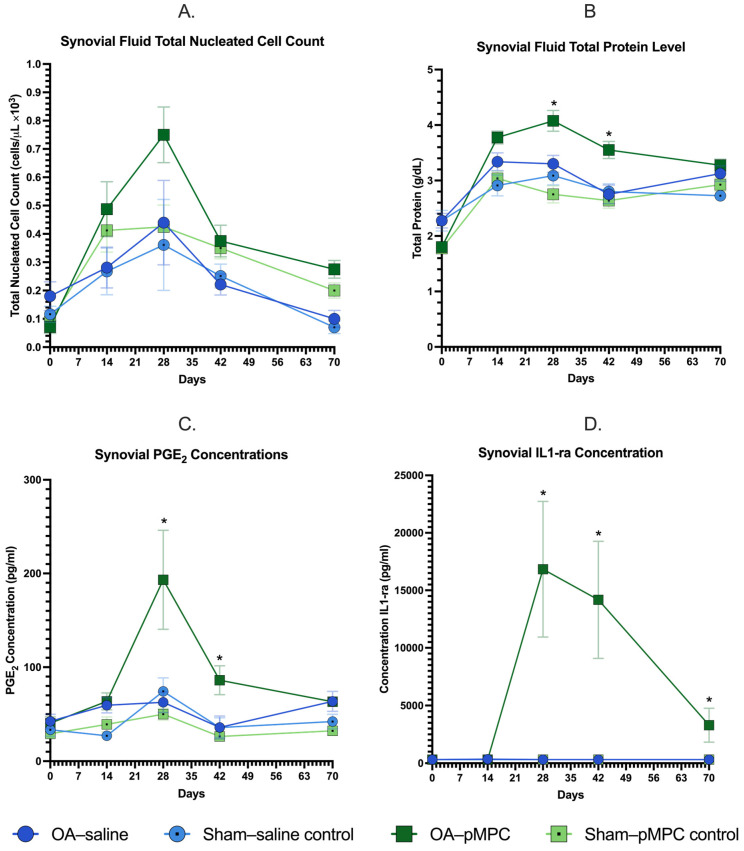
(**A**) Synovial total nucleated cell count, (**B**) Total protein, (**C**) Prostaglandin E2 (PGE_2_), and (**D**) Interleukin 1 receptor antagonist protein (IL-1ra) concentrations. The mean +/− SEM is represented at each evaluation timepoint. Surgery is represented by day 0, and injections were performed on day 14. * represents days synovial concentrations of protein, PGE_2_, or IL1-ra were significantly different for OA–pMPC- and OA–saline-treated joints.

Total nucleated cell counts (TNCC) increased for all joints postoperatively, peaking at d28 (Figure 3A). TNCC was not significantly different for OA–pMPC vs. OA–saline or sham–pMPC vs. sham–saline joints at any time. TNCC populations were primarily monocytes (62–76% for all groups).

#### 3.3.2. Total Protein Concentrations

Total protein concentrations increased for all joints postoperatively (Figure 3B); on days 28 and 42, OA–pMPC joints had significantly higher protein levels than OA–saline joints (4.1 ± 0.5 g/dL versus 3.3 ± 0.4 g/dL, *p* = 0.001 and 3.6 ± 0.4 g/dL versus 2.8 ± 0.5 g/dL, *p* = 0.0008, respectively). There was no difference in total protein concentration between the sham–pMPC control vs. sham–saline control groups.

#### 3.3.3. PGE_2_ Concentrations

PGE_2_ concentrations increased for all joints, peaking at d28 for OA–pMPC joints, and was significantly higher than OA–saline joints for days 28 and 42 (193.22 ± 149.67 pg/mL versus 62.72 ± 30.98, *p* = 0.0006 and 86.18 ± 43.71 versus 35.92 ± 32.04, *p* = 0.005, respectively) (Figure 3C). For OA–pMPC joints, PGE_2_ levels were significantly higher at d28 compared to pre-injection baseline levels d14 (*p* = 0.0008). By d70, values returned to d14 baseline levels. There was no difference in PGE_2_ concentration between the sham–pMPC control vs. sham–saline control groups.

#### 3.3.4. IL-1ra Protein Concentrations

IL-1Ra protein concentrations also peaked at d28 (16,830 ± 15,620 pg/mL versus 312 pg/mL pre-injection d14) for OA–pMPC and remained persistently elevated d28 to 70 (*p* < 0.0001, for each, Figure 3D). All postoperative IL-1ra levels for OA–pMPC were significantly different from pre-injection baseline d14 values (*p* < 0.0001 for days 28 to 70). There was no ifference in IL-1ra concentration between the sham–pMPC control vs. sham–saline control groups.

#### 3.3.5. Glycosaminoglycan Concentrations

GAG concentrations were inversely proportional to PGE_2_ values, with the lowest values at d28 for OA–pMPC, with a gradual return to d14 levels by d70. Values were not significantly different between OA–pMPC and OA–saline at any timepoint, and postoperative values were not significantly different from the d14 baseline. Sham-operated joints were not significantly different from each other and were never significantly different from the d14 baseline.

### 3.4. Serum Biomarkers

Serum biomarkers were not significantly different from each other at any point in time and were never significantly different from d14 baseline values (data in supplemental material).

### 3.5. Diagnostic Imaging

**Figure 4:**
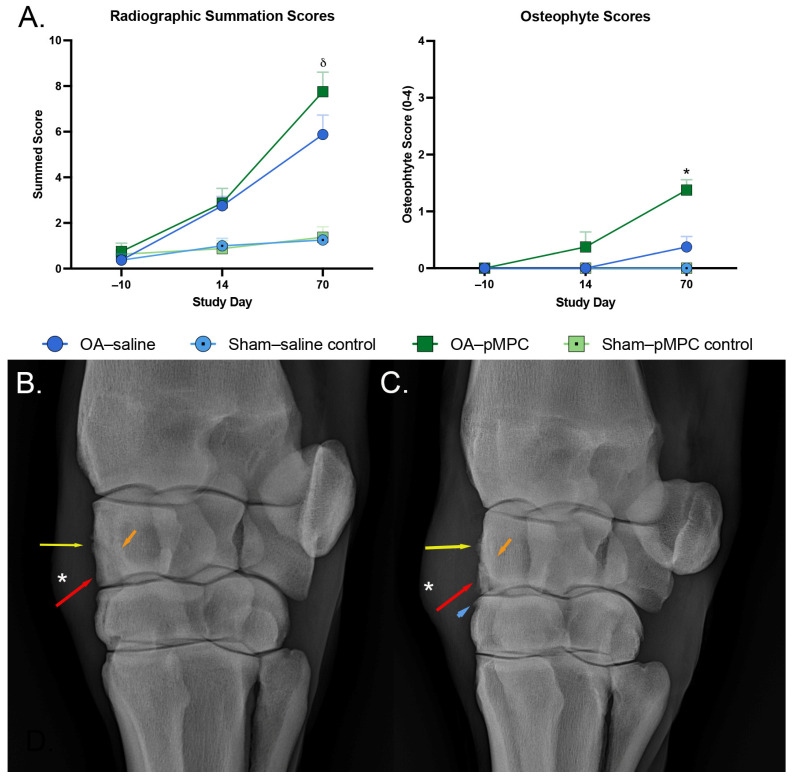
Radiographic examination of both carpi of study horses was performed 10 days prior to the start of the study and again on days 14 and 70 postoperatively. (**A**) Summation scores for all evaluated parameters and osteophyte scores on days −10, 14, and 70. Summation scores were elevated on day 70 for joints with induced OA (δ, *p* < 0.0001); pMPC-treated versus saline-treated summation scores were not significantly different. Osteophyte scores were overall low but significantly higher for OA–pMPC limbs compared to OA–saline limbs on day 70 (*, *p* < 0.0001). (**B**,**C**) Day 70 dorsolateral–palmaromedial radiograph of OA–saline (**B**) and OA–pMPC (**C**); osteochondral fragments (red arrows), sclerosis of the radiocarpal bone (orange arrows), joint capsule enthesopathy (yellow arrows), and joint effusion (white asterisks) were evident in both horses. Osteophytosis (blue arrow) was more prominent in OA–pMPC joints.

Scores for osseous proliferation at the dorsal joint capsule, subchondral bone sclerosis/lysis of RCB and C3, osteophyte formation, and summation scores for all limbs with induced OA increased from day 14 to day 70 (Figure 4A). However, scores between OA–pMPC and OA–saline were not significantly different for any parameters except osteophyte scores (Figure 4). On day 70, osteophyte scores were significantly higher for OA–pMPC limbs compared to OA–saline (*p* = <0.0001). There was no difference in radiographic summation scores nor osteophyte formation between the sham–pMPC control vs. sham–saline control groups. For all joints, osteophyte scores were less than 2 and considered mild.

### 3.6. Gross Pathology and Histology

#### 3.6.1. Gross Pathology

**Figure 5:**
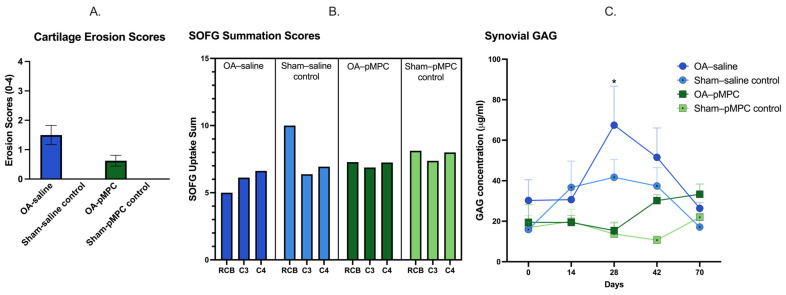
A potential cartilage protective effect by pMPC is highlighted by lower full thickness erosion scores on gross pathology (**A**). Furthermore, GAG concentrations were higher in OA–pMPC joints compared to OA–saline joints although not significantly different (**B**). Synovial GAG concentrations were higher for OA–saline joints compared to OA–pMPC joints on day 28 (*), suggesting that GAG was retained in the cartilage of OA–pMPC joints and not degraded into the synovial fluid (**C**). In (**B**), RCB stands for radiocarpal bone, C3 for third carpal bone, and C4 for fourth carpal bone within the middle carpal joint.

Full thickness, partial thickness, and total erosion scores were higher for untreated horses; however, when comparing OA–pMPC versus OA–saline joints, no significant difference was noticed (Figure 5A). Fragment size, total hemorrhage, the presence of kissing lesions, and synovial adhesions were not significantly different between cohorts nor between OA–pMPC and OA–saline joints. There was no evidence on gross inspection of pMPC within the joint.

#### 3.6.2. Histology—Synovium

Synovial subintimal fibrosis scores were slightly higher for OA–pMPC versus OA–saline joints but not significantly different (*p* = 0.6). Summation scores were identical for OA–pMPC and OA–saline joints and not significantly different. Intimal hyperplasia, subintimal edema, and vascularity were also not significantly different between cohorts nor between joints. There was no evidence of pMPC adjacent to or within the synovial membrane. There were no differences in fibrosis scores between the sham–pMPC control vs. sham–saline control groups.

#### 3.6.3. Histology—Cartilage (RCB, C3, and C4)

Fibrillation scores were low at all locations and not significantly different between OA–pMPC and OA–saline joints nor between sham–pMPC control and sham–saline control joints. Similarly, chondrone formation, chondrocyte necrosis, focal cell loss, and cartilage summation scores were low for all locations and not significantly different. There was no evidence of pMPC in proximity to the cartilage.

#### 3.6.4. Histology—Subchondral Bone (RCB and C3)

Subchondral osteochondral lesions, subchondral bone remodeling, osteochondral splitting, and subchondral summation scores for all locations and all treatment groups were low and not significantly different.

#### 3.6.5. SOFG GAG Content (RCB, C3, and C4)

SOFG uptake was lower for OA joints versus sham-operated joints and lower for OA–saline versus OA–pMPC joints. However, none were significantly different for any location (tangential, intermediate, radiate territorial, radiate interterritorial, and summation; Figure 5B).

### 3.7. Surface Topography

PV, Rq, Ra, and SRz had mean values slightly higher for pMPC-treated versus saline-treated cohorts, with the most notable elevations in OA–pMPC joints. Additionally, Rsk had a slightly lower mean for OA–pMPC joints, indicating that roughness features were skewed towards deeper valleys in those joints. No statistical difference was noticed between any parameter.

## 4. Discussion

We induce mild PTOA by creating an osteochondral fragment and subsequently treat the OA with a single administration of the biolubricant (pMPC) or saline (placebo control). The intra-articular administration of pMPC results in mild cartilage protective effects as indicated by GAG concentrations retained in cartilage; however, the pMPC induces a mild inflammatory state with increases in clinical, synovial, and radiographic scores. While the majority of the experimentally measured parameters are not statistically significant between treatment groups and all parameters are mild, with results being in the bottom quartile, there are some differences between the pMPC and saline-treated joints that should be highlighted. The hypothesis that pMPC decreases physical, radiographic, and biochemical effects in an equine PTOA model is rejected, while gross and histological effects may be interpreted as potentially chondroprotective.

Lameness scores are mild (grade 1 ± 0.5, AAEP scale) for limbs with induced OA. While lameness scores are not significantly different between treatments, lameness scores for OA–pMPC limbs are consistently higher throughout the postoperative study period for both subjective and objective analyses. Vector sum values are above the threshold for detectable clinical lameness for OA–pMPC limbs for the majority of the postoperative period. It should be noted that sham–saline control limbs have elevated lameness scores on days 42 to 63, which could skew OA–saline lameness scores. Two horses in this study group demonstrated elevations in lameness scores on those limbs (evident on the subjective exam). However, the reasons for these lamenesses are unknown. Exclusion of these horses from lameness statistical analysis is considered; however, given the lack of statistical significance, remain in the study. Similar to lameness scores, middle carpal joint effusion and carpal flexion scores remain persistently elevated throughout the postoperative period for OA–pMPC joints. This is not to state that OA–pMPC-treated joints continue to worsen; rather, they do not improve as quickly as OA–saline joints. This is demonstrated by OA–saline joints returning to lameness that is not significantly different from the sham-operated limb by day 21 and day 28 for flexion scores and day 56 for effusion scores. Whereas, OA–pPMC limbs remain persistently elevated through day 28 for lameness scores and for nearly the entire duration of the study for effusion and flexion scores. In sham-operated joints (all received saline), lameness, effusion, and flexion scores for pMPC- and saline-treated horses are similar. Other intra-articular biolubricants [sodium hyaluronan (HA) and polysulfated glycosaminoglycan (PSGAG)], previously assessed in the carpal chip model, demonstrated a slight improvement in lameness scores, effusion scores, and flexion scores from their baseline at 14 days following OA induction when administered three times [28]. Similarly to OA–pMPC-treated joints in the current study, past intra-articular biolubricant treatments have not demonstrated statistically significant improvements over saline in lameness and flexion scores [28]. Multiple PSGAG-treated joints have, however, exhibited significantly improved effusion scores [28]. Hyaluronan sodium chondroitin sulfate and N-acetyl-D-glucosamine combination (PG) in a similar model demonstrate significantly improved average lameness scores for PG-treated joints versus the placebo when administered four times during the study. However, lameness scores for PG versus placebo-treated joints were not significantly different at the end of the study [29]. Effusion scores remain persistently elevated in clinically normal joints with the use of other biolubricants, such as polyacrylamide hydrogels [30]. This result is attributable to the polymer being incorporated into the synovial membrane [30,31]; in this study, there was no evidence of incorporation of the pMPC into the synovial membrane on histological examination.

Synovial fluid analysis partially corroborates a mild inflammatory response. Statistically significant differences between groups are present on days 28 and 42 for synovial total protein, PGE_2_, and IL-1Ra. All synovial parameters peak on day 28, with treatment groups returning to baseline levels by day 70. On day 28, the PGE_2_ levels in OA–pMPC joints are statistically significantly higher than in OA–saline joints (193.22 pg/mL versus 62.72 pg/mL). PGE_2_ levels are known to be significantly elevated in the osteochondral chip model days 7 to 49 postoperatively [33]. Interestingly, these levels are closer to 400 pg/mL for OA-induced joints and 200 pg/mL for joints without chip fracture, with sustained elevations in OA-induced joints [33]. When comparing this result with the total nucleated cell count, the OA–pMPC induces a similar high-normal inflammatory response on the cellular level (800 cells/uL for OA–pMPC joints and 400 cells/uL for OA–saline joints). This degree of inflammatory response is still within reported normal TNCC following elective carpal arthroscopy, with prior reports indicating a range of 500–1250 cells/uL 28 days postoperatively [33,34]. In sham-operated joints, synovial total protein, PGE_2_, and IL-1Ra outcomes are similar to levels present in healthy animals.

IL-1Ra levels markedly increased for the OA–pMPC-treated joints relative to the other groups. Specifically, IL-1Ra concentration spikes on day 28 and then decreases; PGE_2_ levels decrease thereafter, presumably in response to IL-1Ra. In contrast, this peak in PGE_2_ and IL-1Ra is not observed in sham-operated limbs. The pathway for upregulation of endogenous IL-1Ra levels is not clear. However, chondrocytes in humans produce IL-1Ra in response to IL-1β and IL-6 [35,36]. Previous reports demonstrate significant elevations in naturally occurring arthritis, with the highest elevations noted in joints with septic arthritis [37]. The effect of biolubricants on the production of IL-1Ra in horses is not well documented. One study investigated the effects of HA on synovial fluid following arthroscopy for routine osteochondral fragment removal, demonstrating an increase in IL-1Ra at 48 h postoperatively. However, cases were not followed beyond this point [38]. Specifically in this model of carpal PTOA, IL-1Ra elevations following injection of autologous conditioned serum (ACS) result in sustained protein production to approximately 70 pg/mL [39]. In the current study, IL-1Ra levels spike, approaching 25,000 pg/mL. It is unknown why IL-1Ra levels increase so substantially, but it may be in response to an inflammatory state induced by pMPC.

Radiographic studies also reveal persistent mild joint inflammation by evidence of more significant osteophytosis, the likely result of persistent synovitis. Both OA–saline and OA–pMPC joints show radiographic evidence of progressive osteoarthritic change. While the majority of the parameters are not significantly different between OA–saline and OA–PMPC joints, osteophytosis scores are significantly higher in OA–pMPC joints, indicating increased inflammation in these joints.

Interestingly, pMPC treatment of the OA joint reduces synovial GAG release, as evident by the lower concentrations of GAG in the synovial fluid, which may elucidate a protective effect. The differences between groups are largest and statistically significant on day 28, with the release of GAG being the lowest for the pMPC treatment group. At the end of the study, histological GAG scores are higher in cartilage of pMPC-treated joints confirming higher concentrations of GAG remained in the cartilage (Figure 5B), though levels are not significantly different. On gross pathology, total erosion scores for OA–pMPC joints are graded as less than half of the OA–saline counterparts; however, there is no statistical significance. It is possible that a type II statistical error yielded statistical results that are not significant when a true effect is present. The surface topography scores are higher for pMPC-treated joints, indicating higher peaks (increased fibrillation) and lower troughs (cartilage defects/erosions) suggestive of more advanced osteoarthritis. However, scores are not significantly different between saline and pMPC-treated joints. Surface topography, like histology, evaluates a few small portions of cartilage and therefore may not be representative of the entire joint [32].

Previous work on tribological measurements of ex vivo cartilage plugs has demonstrated cartilage protective effects. In the ex vivo study, the pMPC network reduces the coefficient of friction by 73% in cartilage explants compared to saline [9]. It dissipates shear forces at the cartilage interface, thereby reducing damage. Further, due to its network polymer architecture and high hydration due to the presence of phosphorylcholine groups, it also exhibits a “cushioning effect” at the articular surface, which is thought to aid in preventing cartilage damage [9]. In horses, polyacrylamide hydrogels (PAAG) are proposed to function by aggregating on articular cartilage to create a mechanical barrier, resulting in a decrease in the coefficient of friction (COF) by 30–40% relative to saline-treated cartilage explants [23,25].

Histologic grades for synovium, cartilage, and subchondral bone are low and not significantly different between pMPC-treated and saline-treated joints. Gross inspection of the joint and histologic evaluations of synovium and cartilage at the day 70 end point show no evidence of pMPC remaining. While pMPC is resistant to degradation by hyaluronidases and remains in the joint for over 30 days in a murine model, a longer duration of action is not represented here [22]. Another proposed mechanism of action for PAAG in horses is synovial incorporation by macrophages, which improves joint capsule elasticity [23]. The pMPC used in this study does not directly target synovial incorporation as its mechanism of action, and therefore, the effects on synovium are unknown. The information garnered in this study suggests that pMPC exhibits a minimal effect on synovium.

It is unknown why the pMPC initiates an inflammatory response in the joints. In in vitro assays, pMPC over a concentration range of 1 to 100 mg/mL is non-cytotoxic to fibroblasts and chondrocytes over a 72 h incubation period, while at the highest concentration is cytotoxic to synoviocytes. This concentration is significantly higher than that used in the current in vivo study. However, the sustained duration of exposure to the pMPC is greater in vivo.

## 5. Conclusions

The biolubricant used in this study incites a mild inflammatory response intra-articularly and results in increased osteophyte formation; however, it may demonstrate a chondroprotective effect simultaneously. Future research should investigate the effects of different concentrations or formulations of pMPC or doses to minimize toxicity to the synoviocytes while maximizing the lubrication and cushioning properties.

## Data Availability

The raw data supporting the conclusions of this article are contained within the article and/or supplementary material.

## Supplementary Materials

The following are available online at https://www.mdpi.com/article/10.3390/ani15030404/s1.
